# Time to be BRAVE: is educating surgeons the key to unlocking the potential of randomised clinical trials in surgery? A qualitative study

**DOI:** 10.1186/1745-6215-15-80

**Published:** 2014-03-14

**Authors:** Shelley Potter, Nicola Mills, Simon J Cawthorn, Jenny Donovan, Jane M Blazeby

**Affiliations:** 1 Bristol Centre for Surgical Research, School of Social and Community Medicine, University of Bristol, Canynge Hall, 39 Whatley Road, Bristol BS8 2PS, UK; 2 Division of Surgery, Head and Neck, University Hospitals Bristol NHS Foundation Trust, Upper Maudlin Street, Bristol BS2 8HW, UK; 3 Bristol Breast Care Centre, North Bristol NHS Trust, Southmead Hospital, Bristol BS10 5NB, UK

**Keywords:** Breast reconstruction, Education, Methodology, Qualitative, Randomised clinical trials

## Abstract

**Background:**

Well-designed randomised clinical trials (RCTs) provide the best evidence to inform decision-making and should be the default option for evaluating surgical procedures. Such trials can be challenging, and surgeons’ preferences may influence whether trials are initiated and successfully conducted and their results accepted. Preferences are particularly problematic when surgeons’ views play a key role in procedure selection and patient eligibility. The bases of such preferences have rarely been explored. Our aim in this qualitative study was to investigate surgeons’ preferences regarding the feasibility of surgical RCTs and their understanding of study design issues using breast reconstruction surgery as a case study.

**Methods:**

Semistructured qualitative interviews were undertaken with a purposive sample of 35 professionals practicing at 15 centres across the United Kingdom. Interviews were transcribed verbatim and analysed thematically using constant comparative techniques. Sampling, data collection and analysis were conducted concurrently and iteratively until data saturation was achieved.

**Results:**

Surgeons often struggle with the concept of equipoise. We found that if surgeons did not feel ‘in equipoise’, they did not accept randomisation as a method of treatment allocation. The underlying reasons for limited equipoise were limited appreciation of the methodological weaknesses of data derived from nonrandomised studies and little understanding of pragmatic trial design. Their belief in the value of RCTs for generating high-quality data to change or inform practice was not widely held.

**Conclusion:**

There is a need to help surgeons understand evidence, equipoise and bias. Current National Institute of Health Research/Medical Research Council investment into education and infrastructure for RCTs, combined with strong leadership, may begin to address these issues or more specific interventions may be required.

## Background

Patients and surgeons need high-quality information about treatment outcomes to inform decision-making [1]. It is increasingly recognised that well-designed randomised clinical trials (RCTs) provide the best evidence for the effectiveness of an intervention, and, although not suitable for addressing all research questions, they should be the default option for evaluating many, if not all, surgical procedures [2-4]. Surgical trials are difficult to conduct [5], however, and challenges in the implementation of well-designed, pragmatic, multicentre RCTs for surgical procedures have been well-documented [6-8]. Particular challenges relate to the preferences and beliefs of participating surgeons [5,9]. These factors may influence whether a trial is initiated [5], whether participants are recruited effectively [10] and whether the results are accepted and subsequently used to influence practice [3].

A particular challenge is encountered in the effective design and conduct of surgical RCTs in which there are several treatment options and in which patient and surgeon preferences play a key role in procedure selection and patient eligibility. Breast reconstruction (BR) after mastectomy for breast cancer is one such example, where possible interventions include simple implant-based reconstruction, pedicled flap reconstruction including the latissimus dorsi flap, or free flap procedures such as the deep inferior epigastric perforator (DIEP) flap using skin and fat from the lower abdominal wall [11,12]. Since 1995, just 13 BR RCTs [13-24] have been conducted and only 2 have addressed major questions such as the optimal type [24] or timing [25] of surgery. Both of these latter two trials were small, single-centre studies which failed to meet recruitment targets. Systematic reviews have summarised the randomised and nonrandomised evidence for BR surgery [26,27] and highlighted the lack of high-quality research in this area. Although many of the investigators of the nonrandomised studies commented on the limitations of their observational data and the hypothetical need for randomised trials [28-31], prevailing expert opinion suggests that RCTs in BR would be ‘unethical’, ‘impractical’ and/or ‘inappropriate’ [11,28-36] because of the importance of patient and surgeon preference in procedure selection.

These claims and, more broadly, how surgeons’ preferences may influence participation in surgical trials in general have rarely been explored in depth. Qualitative interviews are an excellent method for exploring difficult or sensitive issues [37], such as the basis of surgeons’ preferences, and are increasingly used in the development of trial methodology [9,38]. Our aim in the Breast Reconstruction and Valid Evidence (BRAVE) study was therefore to use qualitative methods to explore surgeons’ preferences and beliefs to gain an understanding of how these factors may influence the feasibility of surgical RCTs using hypothetical trials in BR as a case study.

## Methods

This study received full ethical approval from the Southmead Research Ethics Committee (reference number 09/H0102/50).

### Health-care professional recruitment

Opinion leaders in BR surgery were hypothesised to be key informants regarding the feasibility of randomisation in BR because they would be most likely to participate in any future trial. Our sampling strategy therefore initially targeted surgeons practicing at high-volume centres offering advanced training fellowships.

### Sampling and recruitment

Access to this group of opinion leaders was gained by a committee composed of experienced breast and plastic surgeons and representatives of British professional associations. A 10-minute presentation was made to the group outlining the study, and committee members were invited to participate. Surgeons from high-volume centres that were not represented at the meeting were sent personalised e-mail invitations. Other surgeons with potentially interesting views on randomisation who were recommended by key informants were similarly approached. Colleagues, clinical nurse specialists (CNSs) and psychologists at centres of consenting surgeons were also invited to participate. Nonrespondents were sent an electronic reminder after 3 weeks. Surgeons who did not respond thereafter were considered to have declined study participation.

Maximum variation sampling [39] was initially employed such that male and female surgeons with a range of experience from different geographical and sociodemographic centres across the United Kingdom were initially included [40,41]. As the study progressed, theoretical sampling was introduced to purposively select individuals whose views might add new perspectives to those already represented in an attempt to disprove or contradict emerging theories and allow those theories to be further refined [41,42]. This included seeking deviant or negative cases, such as surgeons who had previously expressed positive views about RCTs in the BR literature or at national meetings. Sampling, data collection and data analysis were conducted concurrently and iteratively [43] and continued until data saturation was achieved and no new themes emerged from the analysis [44].

### Data collection

All interviews were conducted by a female, medically qualified researcher (SP). A semi-structured topic guide for the interviews was developed on the basis of literature reviews and the researcher’s clinical experience (Additional file 1: Study Topic Guides Healthcare Professional Semistructured Interview Schedule for the BRAVE study investigating the feasibility of clinical trials in BR; see Additional file 1).

The topic guide included a series of open questions covering issues related to RCTs in BR to allow participants to direct the discussion, and the questions were iteratively modified as the study progressed to enable exploration of emerging themes in later interviews. All interviews were digitally audio-recorded, transcribed verbatim and anonymised using study pseudonyms. Transcripts were compared with the audio recordings to ensure accuracy. Reflective notes were made after each interview and taken into account in the analysis.

### Data analysis

Data were analysed using a grounded theory approach as described by Glaser and Strauss [45]. The aim of grounded theory is to generate new theories about phenomena that develop from, or are ‘grounded’ in, the data as they are collected and analysed [46]. It is characterised by an inductive–deductive interplay in which sampling, data collection, analysis and theorising occur iteratively or cyclically throughout the study [47]. The central principle of grounded theory is that of constant comparison [48] in which new findings are compared with existing data to identify similarities and differences and emerging theories refined with the ongoing assimilation of the data [46].

Our data analysis was therefore initiated soon after data collection commenced and was an ongoing and iterative process that informed further sampling. The purpose of the analysis was to summarise participants’ views and develop theoretical explanations for their opinions. Interviews were analysed inductively in small batches by systematically assigning codes that captured the meaning of the words to segments of text. Emerging codes were then grouped into similar themes and explored in subsequent interviews. In further analysis, we employed the constant comparison technique of grounded theory [45], in which new findings were compared with existing data to identify similarities and differences within themes, taking into account the context in which these themes were discussed. Emerging theories were developed and refined through ongoing assimilation of the data. This method ensured that findings were systematically compared and grounded in the data. Early codes were subsequently modified and refined, or new codes were added, as the interviews and analyses progressed. The views of individual professionals were compared and contrasted with those of other participants to explore factors which might have influenced attitudes toward RCTs, including profession, surgical speciality, geographical location, experience, gender and previously expressed opinions. Individuals exhibiting contrasting attitudes (‘negative cases’) were studied in detail to gain a deeper understanding of the data. The analysis was conducted by a single researcher (SP) with checking of coding [49] and the plausibility of the data interpretation carried out by an experienced social scientist (NM) and a surgeon (JMB) and by triangulation of health-care professionals’ views [49] to ensure the validity of the data [40,42,44,49-52].

## Results

### Participant demographics

Of 48 invitees, 34 (71%) responded, but 3 interviews could not be arranged. A total of 31 interviews (67% of invitees) at a total of 15 centres were conducted with 11 oncoplastic breast surgeons (OPBSs), 11 plastic surgeons, 11 CNSs (including two interviews with pairs of CNSs) and 2 clinical psychologists. The surgeons performed a median of 30 primary BR operations annually (range, 0 to 290). The majority of surgeons were male (*n* = 17) with a median of 13 years’ experience as consultants (range, 0 to 29 years) (Table 1).

**Table 1:** Demographics of health-care professional study participants

	**Oncoplastic breast surgeons**	**Plastic surgeons**	**Clinical nurse specialists**	**Clinical psychologists**
	**(*n*** **= 11)**	**(*n*** **= 11)**	**(*n*** **= 11)**	**(*n*** **= 2)**
**Gender**				
Male	8	9	0	0
Female	3	2	11	2
**Years as consultant/CNS**^**a **^**experience**				
<5 years	4	1	5	2
6 to 15 years	3	6	6	0
>15 years	4	4	0	0
Median (range)	14 (0 to 23)^b^	13 (4 to 29)	6 (1 to 12)	1 (0 to 1)
**Number of BRs performed per year**				
<20	4^c^	1	NA	NA
20 to 50	3	5		
>50	4	5		
Median (range)	30 (0 to 200)	40 (10 to 290)		

^a^CNS, Clinical nurse specialist; NA, Not applicable. ^b^This group included one senior trainee not yet appointed as a consultant. ^c^This group included two surgeons who did not perform breast reconstruction surgery.

No differences were identified in the demographics of surgeons who participated in the study compared with those who did not. Participants’ perceptions of decision-making and access to care are reported elsewhere [53].

### Participants’ acceptance of randomisation

None of the professionals perceived randomisation to different types of BR surgery (implant, pedicled or free flap) to be an acceptable means of treatment allocation, although the reasons for their strong views were often not clearly articulated and seemed to be overwhelmingly related to their own strong preferences.

“Implants, flap from the back, flap from the tummy, which are the three ways of making a breast as far as I’m concerned—to randomise between those is something that’s largely inappropriate.” (Mr O, plastic surgeon, centre 8)

“When you get appendicitis, you have to have an operation, the doctor tells you, ‘Listen, you need an operation’…. This is completely different. I think the patient has to be involved with the choice.... [T]hey have to be happy with the choice … and feel very comfortable with the decision that’s been made. I think that you’re on a hiding to nothing [have no chance of succeeding] [with randomisation].” (Mr T, plastic surgeon, centre 12

When surgeons reported that the different interventions might have equivalent outcomes, they did agree that randomisation was acceptable; however, they felt that comparisons within specified procedure types (for example, between different types of implants or different forms of lower-pole coverage in implant reconstruction) were more acceptable than comparisons between the very different surgical types of BR (*n* = 23).

“If there were patients in whom there was equipoise an’ in which … one type of reconstruction versus another type would be equally suitable … to randomise those patients, I wouldn’t have a problem … with that.” (Mr G, plastic surgeon, centre 4)

“If we think an abdominal flap is appropriate, you can be randomised between a pedicled TRAM [transverse rectus abdominis myocutaneous] and a DIEP, yeah, I’d be happy with that.... I would be happy with randomising also in other areas where I have equipoise so if I’m able to say this patient is suitable for … an implant and an autologous LD [latissimus dorsi] … or between a TRAM and an implant and a LD [latissimus dorsi pedicled flap], or a TRAM and a DIEP, so that would be combinations of randomisation that I would be happy with, but just a broad randomisation I wouldn’t be happy with.” (Mr C, OPBS, centre 1)

“I am involved in other trials that look at subgroups, and I think that is fair enough because it doesn’t interfere with the basic choice. If you want an implant, then you get an implant, but whether you get implant A or implant B is then probably not as crucial, … and provided the patient had enough information and can give informed consent, then I think that’s a fair enough thing to do.” (Miss W, plastic surgeon, centre 15)

### Understanding participants’ attitudes toward randomisation

Professionals’ acceptance of randomisation was observed to be largely influenced by two factors: (1) their understanding of the limitations of existing data and hence the need for RCTs to generate high-quality evidence in BR and (2) their understanding and acceptance of pragmatic trial design.

#### Limited understanding of problems with existing evidence and need for RCTs in breast reconstruction

A number of professionals did not appear to understand why randomised trials in BR are necessary. This lack of understanding seemed to be due to a limited appreciation of the importance of randomisation in minimising bias. Several professionals said they would refuse randomisation as a method of treatment allocation to determine treatment effectiveness because they perceived that equivalent data could be produced by utilising a number of nonrandomised study designs that included clinical audits.

“I’m telling you how the information that you’re trying to capture from the randomised trial can be captured just by having a good audit.” (Mr J, OPBS, centre 6)

“It is very important that we do produce good long-term outcome data.... [D]o you actually need a randomised controlled trial to produce that data?” (Mr G, plastic surgeon, centre 4)

“We’re always talking about randomised controlled trials…. I would’ve thought, to be honest with you, a far better way is about PROMS [patient-reported outcome measures]…. [I]t’s about the patient-reported outcome measures…. I mean, I think it’s far, far more valuable than a randomised controlled trial.” (Mr T, plastic surgeon, centre 12)

“I just wonder if we need to think a little more laterally about how we assess breast reconstruction…. I know that randomisation is seen as the gold standard, but that’s the gold standard for drugs, not necessarily for surgery, and I just wonder whether there are other ways and other mechanisms that we should be exploring.” (Miss N, OPBS, centre 7)

Most participants who said they felt trials are important were happy to consider randomisation in situations where they believed equipoise to exist:

“There are loads of questions that are not answered. Different manufacturers … one-stage implants as against two-stage, so Becker-35 or a McGhan-150 [different types of implants] against the tissue expander followed by a permanent implant. I would be quite comfortable doing those types of trials because I think they’re equivalent and I really, genuinely don’t know the answer.” (Mr H, OPBS, centre 5)

Randomisation was rejected when equipoise was perceived to be absent. For the majority of professionals, however, perceptions of equipoise were largely based on observational data, personal experience or surgical dogma.

“I’ve got a fairly clear idea of who I think will benefit from [a type of BR].... I would find it difficult to randomise the patients, because my experience has already shown me that they get a better cosmetic outcome.” (Miss N, OPBS, centre 7)

A minority of surgeons recognised this point and hence the need for RCTs to address important questions.

“[I]t’s almost (laughing) a religious fervour that DIEPs are good and pedicled TRAMs are bad, and yet I’m not aware of any good, even-handed evidence to support that.” (Mr H, OPBS, centre 5)

The majority of participants, however, did not appear to appreciate the methodological limitations of the studies on which their perceptions were based or the impact of selection bias on the results. Some surgeons commented that the absence of robust evidence did not equate to the existence of equipoise.

“Theoretically, anywhere where we haven’t got a robust evidence base from randomised trials, you have to say there’s equipoise, but we know that that’s just not the case.” (Mr I, OPBS, centre 6)

A number of surgeons reported that they felt that things had ‘moved on’ in BR and that trials are no longer necessary.

“I’m all for well-designed studies that prove stuff that answers the tricky questions, but, I dunno, I think it’s almost that things have moved on a bit in breast reconstruction…. We’ve now got this toolbox of techniques, and I think it’s now refining those actually and trying to make sure that patients access the right technique for them, I think that’s more the way things should be moving, rather than having two theories that are equal or that seem about the same…. I think we kind of know that.” (Mr O, plastic surgeon, centre 8)

#### Limited understanding and acceptance of pragmatic trial design

Although a number of professionals reported that they felt RCTs are needed, many expressed concerns which reflected limited understanding of pragmatic trial design.

“Each patient is unique—they really are, and it’s [not] like a drug trial where we don’t know the answers. I think there’s so many variables for these patients.” (Mr L, plastic surgeon, centre 6)

“Randomisation … in a nonblinded trial is not as robust as people would like to think it is ’cause it’s heavily affected by patient factors and physician factors, so there’s no doubt, epidemiology is not randomised and it gives you robust outcomes. You wouldn’t need to run a randomised controlled trial of smoking or excessive alcohol intake to convince people that it causes damage. You wouldn’t need to randomise people to two bottles of vodka a day—or not so—to have a robust view about alcohol.” (Mr I, OPBS, centre 6)

A number of surgeons expressed concerns about the complexity of the procedure and the impact it might have on the trial results.

“There are so many variables involved with creating a randomised trial…. Scientifically, you’ll be hounded by people saying that there are so many variables in this that it’s not valid.” (Mr L, plastic surgeon, centre 6)

“The problem with trials is that you have to control for everything…. [I]t’s very easy to set up a trial and find that you’ve actually introduced some bias by something that you’ve done wrong.” (Mr O, plastic surgeon, centre 8)

Many participants were concerned about variations in both the surgeons’ levels of expertise and the procedures themselves and felt that these might invalidate or subvert study findings.

“There’s such a variability that I’m just concerned that say you compare implants and lat [issimus] dorsi’s that you’d be mixing some surgeons who are really not good at lat dorsi’s…., an’ then you’re going to end up saying ‘well the implant gives a nicer result’…. It’s going to skew things unless you have a sort of baseline standardisation of training.” (Miss E, plastic surgeon, centre 3)

“You need to have … [surgeons] who are using exactly the same techniques. Some people change the submammary fold, they lift the thoracoepigastric skin and recreate the sub-mammary fold. Now that’s gonna be totally different from someone who’s keeping the old sub-mammary fold in an immediate reconstruction.” (Mr U, plastic surgeon, centre 14)

Others were concerned about selection bias in a trial:

“By selecting out cases that could go into [the trial], you’re introducing bias … because you’re using your judgement as a clinician to decide who could have one or the other rather than it being a truly randomised process.” (Mr D, OPBS, centre 2)

It’s double-blind randomised controlled trials that are the gold standard, and I don’t see nonblinded, if you like, open-label trials as a gold standard ’cause they’re subject to all sorts of problems, particularly about selection.” (Mr I, OPBS, centre 6)

These concerns appeared to adversely influence professionals’ desire to participate in RCTs that they felt would not generate valid or generalisable results and thus would not impact clinical practice.

## Discussion

Surgical interventions should be evaluated in the context of well-designed, pragmatic, multicentre RCTs because high-quality evidence can guide health policy and clinical decision-making and provide a standard of care for patients considering surgery. Surgical trials are challenging, however, and surgeons’ preferences are an established barrier to their participation and recruitment of patients into trials [7,54]. Our present exploratory, qualitative study including 35 professionals produces further evidence that surgeons do not always accept RCTs or the results. It also provides new data suggesting that surgeons’ rejection of this methodology may be related to their limited understanding of the importance of randomisation in allowing the outcomes of surgical procedures to be fully evaluated, as well as pragmatic trial design issues. Interventions designed to improve professionals’ understanding of the need for trials and pragmatic trial methodology are therefore recommended.

Surgeons’ preferences are a well-recognised and accepted barrier to their participation in trials [10,55-58] but investigators in previous studies have not tried to understand these preferences, how they are formed or the evidence on which they are based, although there is some data to suggest that surgeons’ understanding is limited [56]. By contrast, several studies have explored the basis of patients’ treatment preferences [59-62]. Training research nurse trial recruiters to explore patients’ preferences has been shown to facilitate recruitment of participants into a RCT of watchful waiting, radical radiotherapy and surgery in patients with early prostate cancer [61]. It is possible that such an intervention might be effective in aiding surgeons to recruit patients into RCTs. The limited understanding of pragmatic trial methodology identified in this study is consistent with the findings of a similar previous study conducted with surgeons who had participated in the Spinal Stabilisation Trial (SST) [9], a multicentre, pragmatic RCT comparing an intensive functional rehabilitation programme with surgery for the treatment of chronic lower-back pain. In accordance with the views of the participants in the present study, surgeons recruiting patients into the SST were unsure about the study’s aims or the nature of the comparisons and expressed concerns about the flexible eligibility criteria, which they felt would invalidate the study’s final results. These views had a negative impact on both recruitment during the study and acceptance and interpretation of the study findings. Therefore, a growing body of evidence suggests that surgeons may have a limited understanding of trial methodology.

Although our present study is novel, it may not reflect the true acceptability of RCTs to surgeons, because participants were asked to consider hypothetical trial participation. The similarities between the findings of this study and the SST, however, suggest that the presence of this potential weakness is unlikely. It is also possible that the researcher shaped the study in that, by knowing they were speaking with a physician, the surgeons responded in a particular way. Several strategies were used to ensure the validity of the study data [42,49] and reduce the risk of bias [40,42,44,49-52]. These approaches included triangulation of the health professionals’ views [49], multiple coding of transcripts [49] and checking of data interpretation by the research team. Throughout the study, care was taken to ensure the inclusion of negative cases and the use of ‘fair dealing’, such that multiple differing perspectives have been reported and the viewpoint of only one group has not been presented as the sole truth [42]. Particular attention was paid to the issue of reflexivity and the way in which the researcher, a female medically qualified general surgical trainee (SP) with clinical and research experience with BR, may have shaped the data collection and analysis, as well as how this factor might potentially have influenced the presentation of the results [63]. A clear and comprehensive description of the methods of data collection and analysis have been provided with a range of quotations to illustrate the themes and theories discussed, thus allowing readers to judge for themselves whether the conclusions drawn are adequately supported by the data [42]. Although this study involved surgeons who perform BR surgery, the findings may apply to surgeons in general and may provide novel insights into the ways in which recruitment into surgical trials may be improved.

Well-designed RCTs in surgery are vital to generate high-quality data to guide decision-making, and it is essential that future RCTs be conducted to rigorously evaluate new surgical innovations [2,4] before the procedures are introduced into routine practice. This consideration is of particular importance in operations such as BR, where available procedures differ markedly in cost and evidentiary proof of effectiveness or superiority are necessary to justify diversion of scarce resources to the use of novel techniques while protecting patients from potential harm [64].

RCTs are traditionally unfamiliar to surgeons, however [65]. Unlike drugs, which require robust evaluation before they are approved for use, there are no stringent requirements for the introduction of new procedures in surgery [66-71]. Infrastructure to facilitate high-quality surgical research has also been lacking. There has therefore been little impetus for surgeons to engage in time-consuming and often challenging research [4,5,7,8,72-75], and many early trials failed because of poor recruitment [21]. Embedding qualitative research into surgical trial design is one way in which these problems may be successfully explored and overcome [38,76]. By highlighting previously unanticipated issues relating to a lack of understanding of both the need for RCTs and the role of pragmatic study methodology, however, this study may have identified a potentially novel target for interventions to improve participation in surgical trials: the surgeons themselves.

Integrating education within trials may be one way in which this goal may be achieved [9], but changing the culture of surgical research, creating research capacity and educating the next generation of surgeons about the need for and value of RCTs as a fundamental part of their training may be the most effective long-term strategy for improving recruitment into future surgical trials [5,54]. The Royal College of Surgeons of England recently published a number of recommendations regarding the future of surgical research [77] and initiated plans to create a surgical trials infrastructure through the creation of an Institute of Surgical Research and regional trials units [78]. Integral to these changes is the incorporation of research modules into surgical training [77]. In time, implementation of these recommendations may address some of the issues relating to the lack of familiarity with trial methodology identified in this study and help to ensure that surgical trials are better accepted. An apprenticeship model of ‘see one, do one, teach one’ may be a starting place to change the culture of trial participation by surgeons.

Indeed, there is already evidence that the culture of surgical research in the United Kingdom is changing with the rise of the surgical trainee research collaboratives. The trainee collaborative concept, which originated in the West Midlands has been rapidly disseminated throughout the United Kingdom, and subspecialty collaboratives in orthopaedic and plastic surgery and neurosurgery have also been established [79,80]. The innovative model of trainees at different hospitals working together has been shown to be an effective and efficient means of delivering large, multicentre audit projects [81] and high-quality surgical trials [82]. Trainees are highly motivated to participate in such studies and gain familiarity with research methodology as well as developing valuable practical research skills such as recruiting patients into trials. As such, trainee networks have the potential to build research capacity and make a significant contribution to clinical research [80].

For surgical trainees to be fully engaged with this model, however, changes are required in the way in which research participation and achievements are assessed, both during and at the end of training. The current need to generate academic output for career progression has hindered participation in high-quality clinical research because trainees’ efforts have been diverted into writing up case reports and case series with little awareness of their methodological limitations or toward pursuing more traditional laboratory-based projects in which the required outputs are more easily achieved. Moves are underway to address this issue, and evidence of recruitment of patients into surgical trials and participation in collaborative clinical research, rather than the number of publications, are likely to become minimum future requirements for completion of surgical specialist training.

## Conclusions

There is currently a need for well-designed, pragmatic, multicentre RCTs in many areas of surgery, including BR. At present, however, many professionals do not appear to appreciate the need for RCTs. Furthermore, undertaking a pragmatic RCT is likely to be hampered by surgeons’ limited familiarity with and understanding of this methodology. Interventions to educate surgeons and to allow them to gain familiarity with and confidence in the process may be an essential prerequisite to undertaking such work. Education of the next generation of surgeons by changing the culture of surgical research may be key to unlocking the potential of RCTs in surgery.

## Abbreviations

BR: Breast reconstruction; CNS: Clinical nurse specialist; DIEP: Deep inferior epigastric perforator flap; LD: Latissimus dorsi flap; OPBS: Oncoplastic breast surgeon; PROM: Patient-reported outcome measure; RCT: Randomised clinical trial; TRAM: Transverse rectus abdominis myocutaneous flap.

## Competing interests

The authors declare that they have no competing interests.

## Authors’ contributions

SP conceived of and designed the study, collected and analysed the data and produced the first draft of the manuscript. NM assisted with the study design and data analysis and provided critical input on the drafting of the manuscript. SJC assisted with data collection and the drafting of the manuscript. JD contributed to the conception of the study and the drafting of the manuscript. JMB conceived of and designed the study, commented on the interpretation of the data and assisted in drafting the manuscript. All authors read and approved the final manuscript.

## Supplementary Material

Additional file 1 **Study Topic Guides.** Health-care professional semistructured interview schedule used for the BRAVE study to investigate the feasibility of clinical trials in breast reconstruction. Click here for file
