# Supplementary material for: Time to be BRAVE: is educating surgeons the key to unlocking the potential of randomised clinical trials in surgery? A qualitative study
Source: Trials. 2014 Mar 14;15:80. doi: 10.1186/1745-6215-15-80 (PMC4003809; doi:10.1186/1745-6215-15-80)
Supplement: Additional file 1 — Study Topic Guides. Health-care professional semistructured interview schedule used for the BRAVE study to investigate the feasibility of clinical trials in breast reconstruction. [file 1745-6215-15-80-S1.doc]

Appendix 1 – Study Topic Guides

Healthcare professional semi-structured interview schedule for the BRAVE study investigating the feasibility of clinical trials in breast reconstruction.

Interview to take place at a time convenient to the professional being interviewed.

# Introduction

- Introduce self and research question
- Explain need to record interview and test audio recorder
- Written consent obtained prior to interview – verbally confirm consent to interview and audio recording

# Research questions

1. How do health professionals feel about randomised trials in breast reconstruction and what are the reasons for these feelings?
2. What outcomes (clinical and patient reported) should be evaluated in breast reconstruction research?

# Clarification of details and background information

- Name and age
- Profession and specialty
- Number of years practicing/years of experience
- Types of reconstruction performed at centre
- Numbers of reconstructions performed per year
- Practice of immediate/delayed reconstruction
- Care pathway at centre/how decisions are made

# Randomised trials in breast reconstruction

- Introduce the subject

1. How do you feel about a randomised clinical trial of types of breast reconstruction?
2. Why do you think that? (explore and probe)
3. If a trial was to happen – what study design (if any) would you be happy to participate in and recruit patients to?
4. Are there any reasons why you wouldn’t recruit patients to such a study?
5. Do you think patients would take part in the study? (explore why– yes and no)
6. Barriers and facilitators to recruitment

**Outcome measures and decision making**

Introduce topic and poor quality of outcome reporting to date.

1. What outcomes do you think are important in breast reconstruction?
2. What outcomes are your patients told about during decision making? What evidence is this information based on? If own data – how do they monitor results – database etc?
3. What types of information are patients given? (leaflets, photos, other patients etc)/ PRO/cosmetic/clinical
4. Who is responsible for giving information?
5. What do you think of the quality of the information patients are given? Are the patients satisfied?
6. Do you routinely assess cosmetic outcome? If so, how, if not why? Barriers/facilitators?
7. Do you assess any types of patient reported outcome? Explore why?

# Closing Questions

- Thank them for their time
- Any final comments they would like to make?
